# Influence of Polymorphisms Involved in Platelet Activation and Inflammatory Response on Aspirin-Related Upper Gastrointestinal Bleeding: A Case-Control Study

**DOI:** 10.3389/fphar.2020.00860

**Published:** 2020-06-09

**Authors:** Narmeen Mallah, Maruxa Zapata-Cachafeiro, Carmelo Aguirre, Eguzkiñe Ibarra-García, Itziar Palacios–Zabalza, Fernando Macías-García, J. Enrique Domínguez-Muñoz, María Piñeiro-Lamas, Luisa Ibáñez, Xavier Vidal, Lourdes Vendrell, Luis Martin-Arias, María Sáinz-Gil, Verónica Velasco-González, Adolfo Figueiras

**Affiliations:** ^1^ Department of Preventive Medicine, University of Santiago de Compostela, Santiago de Compostela, Spain; ^2^ Consortium for Biomedical Research in Epidemiology and Public Health (CIBER en Epidemiología y Salud Pública - CIBERESP), Carlos III Health Institute, Madrid, Spain; ^3^ Biocruces Bizkaia Health Research Institute, Pharmacotherapy Group, Bizkaia, Spain; ^4^ University Hospital of Galdakao-Usansolo. Basque Country Pharmacovigilance Unit, Osakidetza, Spain; ^5^ Pharmacology Department, Medicine and Nursing Faculty, University of the Basque Country, Barakaldo, Bizkaia, Spain; ^6^ Osakidetza Basque Health Service, Urduliz Hospital, Pharmacy Department, Urduliz, Spain; ^7^ Department of Gastroenterology and Hepatology, University Hospital of Santiago de Compostela, Santiago de Compostela, Spain; ^8^ Health Research Institute of Santiago de Compostela (IDIS), Santiago de Compostela, Spain; ^9^ Department of Pharmacology, Therapeutics and Toxicology, Catalonian Institute of Pharmacology, Clinical Pharmacology Service, Vall d’Hebron University Teaching Hospital, Autonomous University, Barcelona, Spain; ^10^ Centre for Research on Drug Safety (CESME), Valladolid University, Valladolid, Spain

**Keywords:** aspirin, upper gastrointestinal haemorrhage, genetic polymorphism, pharmacogenomics, platelet, interaction

## Abstract

**Background:**

Despite the wide benefits of aspirin and its cost-effectiveness, aspirin prescriptions have been reduced due to idiosyncratic responses in susceptible individuals. Low-dose aspirin and single-nucleotide polymorphisms (SNPs) are independently associated with increased risk of gastrointestinal hemorrhage; however, to-date, no studies investigated the SNP-aspirin interaction effect on upper gastrointestinal hemorrhage (UGIH). Therefore, we aimed to evaluate the role of 25 SNPs in multiple genes involved in platelet activation, angiogenesis and inflammatory response in aspirin-related UGIH.

**Methods:**

A multicenter, full case–control study was conducted in patients exposed and unexposed to aspirin. Three hundred twenty-six cases diagnosed with UGIH were matched with 748 controls (1:3) by age, gender, health center, and recruitment date. Only adults of European origin were included. Participants were stratified by aspirin exposure and genotype [(Aspirin_(−)_, *wild*-type), (Aspirin_(+)_, *wild*-type), (Aspirin_(+)_, genetic variation), (Aspirin_(−)_, genetic variation)]. For each SNP, the Odds Ratio of UGIH and their 95% confidence intervals were estimated in each subgroup by using the generalized linear mixed models for dependent binomial variables. SNP-aspirin interaction effect was estimated through Relative Excess Risk due to Interaction (RERI) measures.

**Results:**

We observed two categories of SNPs that might modify the risk magnitude of UGIH in aspirin consumers. Seven SNPs (rs1387180 A > G, rs2238631 T > C, rs1799964 T > C, rs5050 T > C/T > G, rs689466 T > C, rs1799983 T > A/T > G, and rs7756935 C > A) were “positive modifiers” associated with an excess of risk from aspirin exposure and carrying that genetic variation (1.75 ≤ RERI ≤ 4.95). On the contrary, the following nine SNPs (rs2243086 G > T, rs1131882 G > A, rs4311994 C > T, rs10120688 G > A, rs4251961 T > C, rs3778355 G > C, rs1330344 C > T, rs5275 A > G/A > T, and rs3779647 C > T) were “negative modifiers” and associated with a reduced risk in aspirin users (−2.74 ≤ RERI ≤ −0.95).

**Conclusion:**

This preliminary study suggests that polymorphisms in genes involved in platelets activity, angiogenesis and inflammatory response might modify the risk of aspirin-related UGIH. Further studies with larger sample size and in different populations are needed to confirm our findings. If confirmed, this might have great impact on public health, thanks to aspirin’s prophylactic properties in diseases of high incidence and severity.

## Introduction

Aspirin is one of the most commonly used medicines worldwide due to its broad spectrum of health benefits such as analgesic, anti-inflammatory and antiplatelet properties (Thorat and Cuzick, 2015). Some recent studies also suggested that aspirin may have protective effects against cancer; a disease that affects all communities and contributes substantially to the global disease burden by impinging on the lives of tens of millions of individuals each year (Global Burden of Disease Cancer Collaboration, 2019). Moreover, there is extensive evidence about the benefits of aspirin use for the secondary prevention against cardiovascular diseases (Godley and Hernandez-Vila, 2016).

On the other hand, patients on aspirin treatment may manifest idiosyncratic reactions to this drug and may be at risk of bleeding, which consequently limit the widespread use of aspirin as prophylactic of many diseases, despite its effectiveness and low cost (Thorat and Cuzick, 2015). Gastrointestinal bleeding is a frequent clinically relevant adverse effect in patients on low-dose aspirin treatment. Several cohort studies and meta-analyses have demonstrated that low-dose aspirin increases the risk of gastrointestinal bleeding between 37% and 85% (Sutcliffe et al., 2013; Whitlock et al., 2015; Whitlock et al., 2016; Raju et al., 2016; Luo et al., 2019; Haykal et al., 2019).

The idiosyncratic response to aspirin could be related to the genetic susceptibility of the individuals. In fact, genetic variations influence patients’ reactions to drugs (Madian et al., 2012). Likewise, several lines of evidence indicated a possible association between predisposing genetic factors and gastrointestinal disorders (Shiotani et al., 2013; Shiotani et al., 2014; Wu et al., 2016; Cho et al., 2016; Milanowski et al., 2017). For instance, previous studies associated the genetic variant rs689466 with an increased risk of ulcerative colitis and gastric cancer, and rs2238631 with upper gastrointestinal hemorrhage (UGIH) in aspirin users (Zhang et al., 2011; Andersen et al., 2011; Wu et al., 2016).

In specific, it was suggested that single-nucleotide polymorphisms (SNPs) mainly those present in genes involved in drug metabolism, platelet activation and inflammatory response, might increase the risk of gastrointestinal bleeding in users of low-dose aspirin (Shiotani et al., 2010). However, the studies were limited by their small sample size and by the assessment of exposed cases, exclusively (Shiotani et al., 2013; Shiotani et al., 2014; Wu et al., 2016; Cho et al., 2016; Milanowski et al., 2017). The non-inclusion of individuals unexposed to aspirin in the design of these studies made it impossible to assess the direct effect of genetic polymorphisms on gastrointestinal bleeding. Therefore, it could not be ascertained whether the previously reported high risk of bleeding in low-dose aspirin users was a consequence of aspirin consumption, the presence of a genetic variation, or a combination of both factors.

Accordingly, in the current “full” case-control study, we aimed at exploring the association between 25 SNPs in genes involved in platelet activation, angiogenesis and inflammatory response and UGIH in a population of exposed and unexposed cases and controls. In addition, we intended to determine any possible interaction or modification of effect between these SNPs and aspirin intake on the risk of upper gastrointestinal bleeding.

## Methods

### Study Population and Study Design

A full case-control study that included 326 cases and 749 controls was conducted. The subjects were adults of at least 18 years of age. They were recruited in two time periods (2004–2007 and 2013–2015) from four health centers located in four different Spanish cities: Barcelona, Galdakao, Santiago de Compostela, and Valladolid. The ethics committee of each involved hospital approved the study protocol and each subject signed a written informed consent before participating in the study.

Cases consisted of hospitalized patients with surgical or endoscopic diagnosis of UGIH. Cases were considered eligible if the diagnosis revealed any of the following ulcers (pyloric, cardia, duodenal, or gastric), erosions (duodenitis pyloric, or cardia), erosive duodenitis, and/or acute gastric mucosal lesions. Hospitalized patients who presented signs of a recent stomach bleeding were also considered as eligible cases, even if the main motive of hospitalization was not UGIH. The signs of recent bleeding were determined according to Forrest classification (Forrest et al., 1974). Cirrhotic patients who did not have variceal esophageal bleeding were included if the endoscopic diagnosis showed any sign of recent bleeding. Patients with endoscopic diagnoses different from those mentioned above were excluded.

Three controls were matched to each case according to their age (+/− 5 years), gender and clinical center. To prevent selection bias due to high aspirin consumption, controls were selected from outpatients or were recruited from the preoperative units among patients who had scheduled non-painful mild surgeries that were not related to aspirin intake. The included surgeries are: prostatic adenoma, prostatic hyperplasia, inguinal or umbilical hernia (strangulated or programmed), eye cataract, phimosis, ear pinning, tubal ligation, plastic surgery, lipoma, vocal cord cyst, septoplasty, varicotomy, thyroid nodules, and thyroglossal cyst (euthyroid). All participants were not biologically related. They were recruited from patients and outpatients of the same health centers in order to ensure that they originate from the same population. Controls that had a history of UGIH or that experienced intrahospital UGIH were excluded. Cases and controls with non-white race were excluded from the analysis in order to control for bias due to population stratification. Participants living outside the study area; having a history of liver cirrhosis or coagulopathy and/or neoplasia; and/or unreliably answered the interview questions were also excluded. A detailed list of exclusion and inclusion criteria is available in supplementary data online (see **Table S1** of the supplementary materials attached to this article).

### Data Collection

A comprehensive questionnaire was designed in a previous study of ours that shares the same protocol (Figueiras et al., 2016). The questionnaire was administered by experienced health personnel who interviewed the subjects. The collected data included: sociodemographic characteristics; smoking, alcohol and caffeine consumption; clinical history; reason for current hospitalization; medicines’ intake; and past episodes of gastric disorders. In addition, cases were asked about the underlying symptomatology, and controls were inquired about the motive of the planned surgery.

Complete pharmacological anamnesis was generated using the following four complementary strategies. First, direct questions were raised to the patients about the name, the daily dose, the indication of use and the source of prescription of the medicines consumed in the past two months, including over-the-counter drugs. Second, information was obtained about the frequent symptoms for which aspirin was prescribed, and the treatments used to reduce these symptoms. Third, prompt cards of the most common commercial aspirin boxes were shown to the participants in order to facilitate the recall process. Fourth, subjects who could not remember a specific information were re-interviewed later during their hospitalization, or were telephoned in case they were discharged before repeating the interview. When a patient was in a poor health condition and could not answer the whole interview, the accompanying persons who were in charge of the patient’s medication (healthcare assistants or direct relatives) were allowed to help him/her complete the interview, however only the information confirmed by the patient was considered. In case the patient had doubts or was not certain about the answers, the given information was confirmed by reviewing the medical records of the patient. Moreover, patients who doubted about the name or the dose of the consumed medicine were telephoned once they discharged from the hospital and requested to check this information in the corner drugstore.

At the end of the interview, the interviewer rated the perceived reliability of each interview by using 0–10 Likert scale. Interviews with a zero score were considered completely unreliable and excluded from the analysis. The scores of the included interviews were then inspected for their influence on the estimated measure of effect as explained later in the statistical analysis section.

An index date of aspirin intake was established in order to ensure that exposure happened prior to any symptom of UGIH. For the cases, the index date was set as the day of occurrence of the first signs or symptoms of UGIH. For the controls, the index date was defined as the interview date. In line with earlier studies, we considered an etiological window of 7 days from the index day. Aspirin intake that occurred after the index date was not considered as exposure in the statistical analysis.

### Determination of *Helicobacter pylori* (*H. pylori*) Infection

The presence of anti–*H. pylori* immunoglobulin G was tested in human serum using the Enzyme Linked Immunosorbent Assay (ELISA) commercial kits [Human Anti-Helicobacter pylori IgG ELISA Kit (ab108736, Abcam, Cambridge, England), and Captia™ H. pylori IgG EIA (ref: 2346400, Trinity Biotech Captia, Co. Wicklaw, Ireland)] according to the manufacturer’s protocol. To avoid obtaining any false positive result due to an old infection, patients were asked whether they had been previously treated against *H. pylori* infection.

### Selection of SNPs and Genotyping

An extensive literature review was carried out until April 2017 in order to identify SNPs associated with gastrointestinal disorders, the function of the corresponding genes and the clinical significance of the genetic variation. Subsequently, related SNPs were selected, and samples were genotyped using the iPlex^®^ Gold chemistry and MassARRAY platform, according to manufacturer’s instructions (Agena Bioscience, San Diego, EEUU). Genotyping assays were designed using the Agena Bioscience MassARRAY Assay Designer 4.1 software and were performed in 384-well plates. The quality of the genotyping was checked by including negative controls and a trio of Coriell samples. In addition, the reproducibility of 7% of the samples was tested between and/or within plates. Finally, all clusters plots were checked manually by trained personnel using MassArray Typer software.

Possible bias in the selected controls was assessed by checking the compliance of the genotyped SNPs with Hardy-Weinberg equilibrium (HWE). HWE test was performed using the SNPassoc Library of the R software package (Version 1.9-2).

### Statistical Analysis

The participants were stratified according to aspirin exposure [aspirin(+); aspirin(−)] and to genetic profile of each of the 25 SNPs (*wild-type*; genetic-variation). Accordingly, for each SNP, the patients were grouped into four categories [aspirin(−); *wild-type*], [aspirin(+); *wild-type*], [aspirin(−); genetic variation] and [aspirin(+); genetic variation]. The adjusted Odds Ratios (ORs) of UGIH were calculated in each group in comparison with the category [aspirin(−); *wild-type*]. Subsequently, any potential interaction effect upon the co-presence of genetic polymorphisms and aspirin exposure was explored by estimating the Synergism index (S) (also called effect modification) and the Relative Excess Risk due to Interaction (RERI) along with their 95% CI.

The ORs and their 95% confidence intervals (CI) were estimated using the generalized linear mixed models for dependent binomial variables (González et al., 2014). To build up the statistical models, the following four levels were considered consecutively: study subjects, strata (each case and its three matched controls), recruitment center, and period of patients’ enrolment.

A bivariate analysis was carried out to test the effect of the potentially confounding variables. Covariables with a P-value < 0.2 was selected for multivariate logistic regression analysis, while those with higher levels of statistical significance were eliminated successively from the original model, provided that the coefficients of the main exposure variables did not change by more than 10%, and that Schwartz’s Bayesian Information Criterion was improved (Schwarz, 1978; Mickey and Greenland, 1989; Bates et al., 2015). The covariables that were retained in the final model are: age, Body Mass Index (BMI), gender, history of arthrosis, Helicobacter Pylori infection, gastrointestinal disorders (ulcer or bleeding), source of information (patients or health assistants/direct relatives), number of conducted interviews, answers’ reliability score, and exposure to each of the following medicines: Nonsteroidal Anti-Inflammatory Drugs (NSAIDs) except aspirin, inhibitors of the proton pump, antiaggregant and anticoagulants. The models were estimated using the lmer function of the lme4 R package (version 1.1-21) (Brown and Prescott, 2006). The analysis was restricted to individuals with complete data on the variables included in the model. The recommendations of Knol M.J, 2012 were followed to represent the interaction between aspirin and each SNP (Knol and VanderWeele, 2012). S was obtained from the ratio of the combined effects to the sum of each of the individual effects of aspirin and polymorphisms. RERI was estimated by contrasting the effects of aspirin exposure and polymorphism together to the sum of each factor considered separately: RERI = OR[(genetic variation; aspirin(+)] – OR[(*wild-type;* aspirin(+)] – OR[(genetic variation; aspirin(−)] +1 (Hosmer and Lemeshow, 1992; Andersson et al., 2005). The confidence intervals of the interaction terms were calculated by applying the method developed by Figueiras et al. (Figueiras et al., 1998).

## Results

### Study Population

Of 5,896 interviewed subjects, 326 cases and 748 controls were eligible to be included in the final analysis. The most common exclusion criteria were ineligible endoscopic diagnosis (1,590 cases) and unavailability of biological material (328 cases and 748 controls). The flow diagram of cases and controls enrollment in the study and the reasons of exclusion are presented in supplementary data online (see **Figure S1** and **Table S1** of the supplementary materials attached to this article). **Table 1** summarizes the demographic and clinical characteristics of the study population.

**Table 1:** Description of cases and controls.

Characteristic	Cases (N = 326) № (%)	Controls (N = 748) № (%)
**Age**		
<45	41 (12.6%)	95 (12.7%)
45–65	117 (35.9%)	271 (36.2%)
>65	161 (49.4%)	370 (49.5%)
missing	7 (2.1%)	12 (1.6%)
**BMI**		
Underweight	10 (3.1%)	24 (3.2%)
Normal weight	114 (35.0%)	204 (37.3%)
Overweight	128 (39.3%)	374 (50.0%)
Obese	68 (20.9%)	144 (19.3%)
Missing	6 (1.8%)	2 (0.3%)
**Gender**		
Male	236 (72.4%)	559 (74.7%)
Female	87 (26.7%)	189 (25.3%)
Missing	3 (0.9%)	0
**Arthrosis**		
No	219 (67.2%)	469 (62.7%)
Yes	86 (26.4%)	216 (28.9%)
Missing	21 (6.4%)	
***Helicobacter pylori***		
No or inconclusive	27 (8.3%)	138 (18.4%)
Yes	276 (84.7%)	574 (76.7%)
Missing	23 (7.1%)	36 (4.8%)
**Source of information**		
Patients	259 (79.4%)	672 (89.8%)
Healthcare assistants/direct relatives	67 (20.6%)	76 (10.2%)
**Interview Variables**		
**Number of interviews conducted**		
1	274 (84.0%)	644 (86.1%)
≥2	52 (16.0%)	104 (13.9%)
**Reliability of the interview**		
<5	13 (4.0%)	20 (2.7%)
5–7	36 (11%)	78 (10.4%)
7–9	134 (41.1%)	310 (41.4%)
≥9	143 (43.9%)	340 (45.5%)
**Personal history of gastrointestinal disorders**		
None or dyspepsia	208 (63.8%)	647 (86.5%)
Ulcer	48 (14.7%)	56 (7.5%)
Bleeding	70 (21.5%)	45 (6.0%)
**Exposure to other medications**		
All Nonsteroidal anti-inflammatory drugs (NSAIDs)	131 (40.2%)	112 (15.0%)
All NSAIDs except aspirin	111 (34.0%)	96 (12.8%)
NSAIDs metabolized by CYP2C9	58 (17.8%)	37 (4.9%)
NSAIDs (M01A group)	74 (22.7%)	48 (6.4%)
Inhibitors of COX2	3 (0.9%)	6 (0.8%)
Nonsteroidal anti-inflammatory agents	1 (0.3%)	1 (0.1%)
Aspirin and its derivatives	32 (9.8%)	19 (2.5%)
Analgesics not narcotics	54 (16.6%)	56 (7.5%)
Inhibitors of the proton pump	36 (11.0%)	66 (8.8%)
Antiaggregant	65 (19.9%)	86 (11.5%)
Anticoagulants	35 (10.7%)	32 (4.3%)

### Single-Nucleotide Polymorphisms Genotyping

All the 1,074 biologically unrelated patients were genotyped. High quality of genotyping testing was obtained as revealed by the reproducibility analysis (100%) and genotype recall rate (≥98%). In addition, the controls were in equilibrium with respect to the corresponding SNPs as confirmed by the manual inspection of the clusters’ plots and the HWE analysis (p < 0.001) (see **Table S2** of the supplementary materials attached to this article).

### Risk Estimation and Interaction Analysis

Aspirin consumption substantially increases the risk of occurrence of UGIH by around six folds [adjusted OR: 5.82 (95% CI: 2.2–10.08)].

Stratifying the analysis by genotype (*wild*-type vs. genetic variation) and aspirin exposure yielded the following two distinct groups of SNPs. The first group comprises SNPs that increased the risk magnitude of aspirin-related UGIH and was called “positive modifiers”. The second group consists of SNPs that showed protective effect by decreasing the risk value and was named “negative modifiers”.

### SNPs as Positive Modifiers of the Effect of Aspirin-Related UGIH

In the absence of aspirin exposure, no association was observed between carrying the following genetic variations and the occurrence of UGIH (rs1387180 A > G, rs2238631 T > C, rs1799964 T > C, rs5050 T > C/T > G, rs689466 T > C, rs1799983 T > A/T > G, and rs7756935 C > A) (**Table 2**). However, when exposed to aspirin, carriers of the *wild*-type genotypes of these SNPs are at lower risk of occurrence of UGIH in comparison with carriers of the corresponding genetic variations. The estimated effect on the additive scale of aspirin use by carriers of these genetic variations is larger than the effect of aspirin use by carriers of the *wild*-type genotypes. This was also revealed by RERI estimations that indicates the presence of positive effect modification of aspirin use across the strata of genotypes (1.75 ≤ RERI ≤ 4.95), though these estimations were not statistically significant (**Table 2**).

**Table 2:** Odds ratios (OR) for upper gastrointestinal bleeding associated with aspirin intake and SNPs of the positive modifiers category, and their interaction assessment represented by Synergism Index (S) and Relative Excess Risk due to Interaction (RERI).

Single-Nucleotide Polymorphism (Reference number)	Wild-type genotype		Genetic Variation		OR* (95% CI) for any genetic variation within strata of aspirin intake; p-value	RERI (95% CI)	S (95% CI)
	N cases/controls	OR* (95% CI); p-value	N cases/controls	OR* (95% CI); p-value			
**rs1387180 A > G** Aspirin intake (No)	143/358	1.00	119/278	0.84 (0.60–1.18) P = 0.3233	0.84 (0.60–1.18) p = 0.3278	4.28 (−4.41–12.97)	3.33 (0.4–27.59)
Aspirin intake (Yes)	10/12	2.99 (1.07–8.37) P = 0.0369	14/5	7.12 (2.22–22.83)	3.26 (0.44–29.51) P = 0.2298		
OR (95% CI) for Aspirin intake within strata of genotype; p-value		3.19 (1.12–9.08) P = 0.0293		8.74 (2.61–29.24) P = 0.0001			
**rs2238631 T > C** Aspirin intake (No)	74/168	1.00	188/468	0.97 (0.67–1.41) P = 0.8765	0.97 (0.67–1.42) P = 0.8932	2.22 (−4.17–8.61)	2.08 (0.18–24.13)
Aspirin intake (Yes)	6/5	3.08 (0.69–13.81) P = 0.1414	18/12	5.28 (2.12–13.14) P = 0.0004	1.57 (0.17–14.97) P = 0.6935
OR (95% CI) for Aspirin intake within strata of genotype; p-value		3.78 (0.82–17.43) P = 0.0881		5.44 (2.25–13.17) P = 0.0002	
**rs1799964 T > C** Aspirin intake (No)	150/372	1.00	112/264	1.19 (0.84–1.66) P = 0.3251	1.19 (0.84–1.66) P = 0.3265	3.29 (−5.33–11.91)	2.13 (0.36–12.73)
Aspirin intake (Yes)	12/10	3.73 (1.34–10.40) P = 0.0120	12/7	7.20 (2.38–21.79) P = 0.0005	2.17 (0.35–13.38) P = 0.4027		
OR (95% CI) for Aspirin intake within strata of genotype; p-value		3.86 (1.36–10.92) P = 0.011		5.73 (1.84–17.81) P = 0.0026			
**rs5050 T > C/T > G** Aspirin intake (No)	85/188	1.00	177/448	0.82 (0.57–1.18) P = 0.2765	0.82 (0.57–1.18) P = 0.2746	1.75 (−4.19–7.71)	1.84 (0.22–15.6)
Aspirin intake (Yes)	9/8	3.26 (1.03–10.34) P = 0.0444	15/9	4.83 (1.72–13.60) P = 0.0028	1.22 (0.22–6.90) P = 0.819		
OR (95% CI) for Aspirin intake within strata of genotype; p-value		2.75 (0.78–9.73) P = 0.1174		6.05 (2.22–16.53) P = 0.0004			
**rs689466 T > C** Aspirin intake (No)	170/393	1.00	92/243	0.85 (0.60–1.20) P = 0.3583	0.84 (0.59–1.20) P = 0.3431	4.95 (−6.39–16.26)	3.18 (0.43–23.29)
Aspirin intake (Yes)	16/12	3.42 (1.40–8.36) P = 0.0069	8/5	8.22 (2.14–31.59) P = 0.0022	2.98 (0.37–23.96) P = 0.3036		
OR (95% CI) for Aspirin intake within strata of genotype; p-value		3.72 (1.49–9.26) P = 0.0048		7.68 (2.01–29.36) P = 0.0029			
**rs1799983 T > A/T > G** Aspirin intake (No)	126/302	1.00	136/336	1.28 (0.92–1.80) P = 0.1462	1.29 (0.92–1.81) P = 0.1361	3.40 (−5.91–12.72)	2.03 (0.36–11.6)
Aspirin intake (Yes)	14/10	4.01 (1.43–11.20) P = 0.0081	10/7	7.69 (2.49–23.74) P = 0.0004	4.42 (0.54–36.33) P = 0.1665		
OR (95% CI) for Aspirin intake within strata of genotype; p-value		4.13 (1.35–12.57) P = 0.0126		4.89 (1.62–14.77) P = 0.0048			
**rs7756935 C > A** Aspirin intake (No)	150/347	1.00	112/289	1.02 (0.72–1.44) P = 0.9239	1.02 (0.73–1.44) P = 0.9012	1.84 (−5.70–9.38)	1.61 (0.25 – 10.17)
Aspirin intake (Yes)	14/9	4.02 (1.48–10.93) P = 0.0064	10/8	5.88 (1.90–18.21) P = 0.0022	1.82 (0.31–10.85) P = 0.5091		
OR (95% CI) for Aspirin intake within strata of genotype; p-value		3.86 (1.42–10.50) P = 0.0081		6.67 (2.11–21.62) P = 0.0013			

(*): Odds Ratio adjusted for: period of patients´ recruitment, previous history of arthrosis, infection with Helicobacter pylori, gastrointestinal disorders (ulcer and bleeding), exposure to NSAIDs except ASA, exposure to inhibitors of the proton pump, exposure to antiaggregant, exposure to anticoagulants, and the interview variables (the number and the reliability of the interview).

### SNPs as Negative Modifiers of the Effect of Aspirin-Related UGIH

Furthermore, carrying any of the following nine SNPs was not associated with the risk of UGIH when the patients were not exposed to aspirin [rs2243086 G > T, rs1131882 G > A, rs4311994 C > T, rs10120688 G > A, rs4251961 T > C, rs3778355 G > C, rs1330344 C > T, rs5275 A > G/A > T, and rs3779647(C > T)] (**Table 3**). However, on the contrary to the pattern of association observed earlier, when exposed to aspirin, carriers of the *wild*-type genotypes of these SNPs are at higher risk of occurrence of UGIH in comparison with carriers of the corresponding genetic variations.

**Table 3:** Odds ratios (OR) for upper gastrointestinal bleeding associated with aspirin intake and SNPs of the negative modifiers category, and their interaction assessment represented by Synergism Index (S) and Relative Excess Risk due to Interaction (RERI).

Single-Nucleotide Polymorphism (Reference number)	Wild-type genotype		Genetic Variation		OR* (95% CI) for any genetic variation within strata of aspirin intake; p-value	RERI (95% CI)	S (95% CI)
	N cases/controls	OR* (95% CI); p-value	N cases/controls	OR* (95% CI); p-value			
**rs2243086 G > T** Aspirin intake (No)	172/403	1.00	89/232	0.96 (0.67–1.36) P = 0.7987	0.95 (0.67–1.35) P = 0.7811	−0.95 (−7.84–5.94)	0.76 (0.1–5.99)
Aspirin intake (Yes)	18/12	5.02 (2.01–12.54) P = 0.0005	6/5	4.03 (1.07–15.23) P = 0.0398	0.24 (0.03–2.18) P = 0.2029		
OR (95% CI) for Aspirin intake within strata of genotype; p-value		5.41 (2.12–13.83) P = 0.0001		3.71 (0.94–14.65) P = 0.0615			
**rs1131882 G > A** Aspirin intake (No)	183/431	1.00	79/205	0.97 (0.68–1.40) P = 0.8778	0.97 (0.67–1.40) P = 0.8705	−2.74 (−9.42–3.94)	0.43 (0.05–3.69)
Aspirin intake (Yes)	18/10	5.86 (2.26–15.17) P = 0.0003	6/7	3.09 (0.87–10.93) P = 0.0798	0.01 (0.00–0.54) P = 0.0264		
OR (95% CI) for Aspirin intake within strata of genotype; p-value		5.68 (2.18–14.81) P = 0.0001		4.69 (1.19–18.55) P = 0.0276			
**rs4311994 C > T** Aspirin intake (No)	190/454	1.00	72/182	0.97 (0.67–1.40) P = 0.8688	0.96 (0.66–1.40) P = 0.8472	−1.78 (−8.42–4.87)	0.58 (0.06–5.46)
Aspirin intake (Yes)	18/12	5.25 (2.15–12.82) P = 0.0003	6/5	3.44 (0.84–14.07) P = 0.0855	0.24 (0.01–4.57) P = 0.3399		
OR (95% CI) for Aspirin intake within strata of genotype; p-value		5.40 (2.21–13.22) P = 0.0002		3.56 (0.80–15.92) P = 0.096			
**rs10120688 G > A** Aspirin intake (No)	135/305	1.00	127/331	0.94 (0.68–1.32) P = 0.7369	0.95 (0.68–1.32) P = 0.7454	−2.76 (−11.41–5.90)	0.49 (0.07–3.35)
Aspirin intake (Yes)	10/5	6.52 (1.88–22.54) P = 0.0031	14/12	3.70 (1.41–9.71) P = 0.0078	0.44 (0.07–2.95) P = 0.4018		
OR (95% CI) for Aspirin intake within strata of genotype; p-value		5.98 (1.73–20.63) P = 0.0047		4.94 (1.77–13.73) P = 0.0022			
**rs4251961 T > C** Aspirin intake (No)	115/305	1.00	147/331	1.20 (0.86–1.69) P = 0.2780	1.21 (0.86–1.69) P = 0.2751	−2.59 (−10.37–5.18)	0.54 (0.09–3.35)
Aspirin intake (Yes)	13/8	6.43 (2.30–18.03) P = 0.0004	11/9	4.05 (1.32–12.39) P = 0.0144	0.52 (0.09–2.90) P = 0.4543		
OR (95% CI) for Aspirin intake within strata of genotype; p-value		6.22 (2.19–17.68) P = 0.0006		3.25 (1.04–10.08) P = 0.0418			
**rs3778355 G > C** Aspirin intake (No)	133/326	1.00	129/3100	1.0 (0.72–1.40) P = 0.9998	1.01 (0.72–1.40) P = 0.9752	-1.26 (-8.28–5.75)	0.71 (0.11–4.56)
Aspirin intake (Yes)	13/8	5.38 (1.85–15.65) P = 0.0020	11/9	4.12 (1.41–12.00) P = 0.0095	0.67 (0.11–4.17) P = 0.6697		
OR (95% CI) for Aspirin intake within strata of genotype; p-value		5.80 (1.95–17.26) P = 0.0016		3.93 (1.35–11.44) P = 0.0121			
**rs1330344 C > T** Aspirin intake (No)	160/389	1.00	102/247	1.09 (0.78–1.54) P = 0.6050	1.10 (0.78–1.55) P = 0.5802	-1.44 (−8.41–5.54)	0.68 (0.09–4.98)
Aspirin intake (Yes)	15/12	5.36 (2.13–13.49) P = 0.0004	9/5	4.02 (1.11–14.54) P = 0.0341	0.66 (0.11–3.83) P = 0.6408		
OR (95% CI) for Aspirin intake within strata of genotype; p-value		5.38 (2.11–13.75) P = 0.0004		3.42 (0.90–12.96) P = 0.0706			
**rs5275 A > G/A > T** Aspirin intake (No)	119/292	1.00	129/338	1.0 (0.71–1.40) P = 0.9964	1.0 (0.71–1.41) P = 0.9966	−1.66 (−9.93–6.62)	0.65 (0.09–4.71)
Aspirin intake (Yes)	7/6	5.72 (1.53–21.33) P = 0.0094	15/10	4.06 (1.54–10.73) P = 0.0047	0.24 (0.02–2.49) P = 0.2307		
OR (95% CI) for Aspirin intake within strata of genotype; p-value		6.31 (1.71–23.24) P = 0.0056		4.33 (1.62–11.59) P = 0.0036			
**rs3779647 (C > T)** Aspirin intake (No)	129/323	1.0	133/313	1.07 (0.77–1.49); p = 0.6930	1.07 (0.77–1.5); p = 0.6733	-1.57 (-8.56, 5.43)	0.66 (0.1–4.41)
Aspirin intake (Yes)	13/11	5.53 (2.08–14.69); p = 0.0006	11/6	4.03 (1.24–13.12); p = 0.0205	0.67 (0.11–4.24); p = 0.6722		
OR (95% CI) for Aspirin intake within strata of genotype; p-value		6.10 (2.27–16.43); p = 0.0003		3.61 (1.10–11.87); p = 0.0345			

(*): Odds Ratio adjusted for: period of patients´ recruitment, previous history of arthrosis, infection with Helicobacter pylori, gastrointestinal disorders (ulcer and bleeding), exposure to NSAIDs except ASA, exposure to inhibitors of the proton pump, exposure to antiaggregant, exposure to anticoagulants, and the interview variables (the number and the reliability of the interview).

These findings would indicate that the estimated effect on the additive scale of aspirin use by carriers of these genetic variations is smaller than the effect of aspirin use by carriers of the corresponding *wild*-type genotypes. The interaction analysis revealed that there is negative effect modification of aspirin use across the strata of genotypes on an additive scale (−2.74 ≤ RERI ≤ −0.95); however, this modification was not statistically significant.

Finally, no modification of effect was observed for the SNP rs2990510 T > G [OR *_wild_*_-type_: 3.98 (95% CI: 1.24–12.76) vs. OR _genetic-variation_: 4.26 (95% CI: 1.58–11.47); *S*: 1.19 (95% CI: 0.15–9.42); RERI: 0.51 (95% CI: −5.58, 6.61)]. In addition, no conclusive interpretation could be done about the following 8 SNPs due to the limited number of cases and controls who were aspirin users and carriers of these genetic variations (< 5 subjects in one of the groups): rs2502488, rs1800629, rs361525, rs1143627, rs16944, rs3842787, rs3842788 and rs5788 (see **Table S3** of the supplementary materials attached to this article).

## Discussion

To our knowledge, this is the first and largest case-control study that assesses the effect of a large number of SNPs in multiple genes involved in platelet aggregation angiogenesis and inflammatory response on the risk of aspirin-related UGIH, and that explores the excess of risk from SNPs-aspirin interaction. We tested the presence of genetic variations at the DNA sequence level, and our preliminary data suggested that variations in these genes might alter the risk magnitude of UGIH in aspirin users. These results are of a high clinical interest as they indicate that the likelihood of occurrence of the idiosyncratic bleeding response to aspirin depends on the patient’s genotype.

We identified a group of seven SNPs that act as “positive modifiers” by increasing the risk magnitude of UGIH in aspirin users up to more than three folds. Among these SNPs, rs1387180 A > G, rs2238631 T > C, and rs689466 T > C yielded the greatest excess of risk. A hypothesis that could explain these effects is that SNPs belonging to this group “positive modifiers” might cause a reduction in platelets’ activity, and therefore contribute to increasing the risk of bleeding. Platelets play a fundamental role in homeostasis and platelet rich plasma are suggested to treat specific hemorrhages such as hemorrhagic cystitis (Masieri et al., 2019). In fact, the genetic variant rs1387180 was associated earlier with decreased platelets activity in diabetic patients on aspirin treatment (Postula et al., 2013). rs689466 belongs to COX-2, a gene involved in the production of prostaglandins which plays a role in platelets aggregation and protects against gastric damage (Caughey et al., 2001). Our findings are in line with previous studies which associated rs689466 with an increased risk of gastrointestinal disorders such as ulcerative colitis and gastric cancer (Andersen et al., 2011; Zhang et al., 2011). We also coincide with Yun Wu who reported that rs2238631 is a risk factor of upper gastrointestinal bleeding in aspirin users (Wu et al., 2016). Nevertheless, our results go beyond that of Wu by showing that aspirin users who are carriers of the *wild*-type genotype of rs2238631 are not at significant risk of UGIH, and that this genetic variation presents an additional risk of bleeding.

We also detected another group of nine SNPs “negative modifiers” that were found to decrease the risk magnitude of aspirin-related UGIH. As opposed to the “positive modifiers” SNPs, the genetic variants in this category might have a role in enhancing platelets activity which would therefore aid in lowering the odds of occurrence of bleeding. In this regard, an association between rs1131882, rs4311994 and rs3779647 and increased platelet activity in diabetic patients on aspirin therapy has already been reported (Postula et al., 2011; Postula et al., 2013). Studies also indicated that the presence of genetic variations in the COX-2 gene such as rs5275 boosts COX-2 expression and therefore aid in reducing the risk of myocardial infarction and ischemia; cardiac conditions in which platelet activation makes an important contribution (Haybar et al., 2018). rs4251961 was associated with higher expression of fibrinogen that plays a key role in hemostasis, and thus participates in the prevention of bleeding (Wassel et al., 2011). In addition, Postula and colleagues, reported that rs10120688 is associated with decreased platelets activity in aspirin users (Postula et al., 2011). In this study, we also found that aspirin users who are carriers of rs10120688 are at an increased risk of UGIH. However, additionally, we showed that aspirin consumers who are carriers of the *wild*-type of rs10120688 are at higher risk of developing UGIH than the consumers who are carriers of the genetic variation.

In a similar fashion, previous reports also suggested that rs2243086, rs3778355 and rs1330344 are possible risk factors of gastric mucosal injury induced by aspirin (Wu et al., 2016). In the present study, we also detected that carriers of these genetic variations are at increased odds of developing UGIH, and found that the risk magnitude is even higher in carriers of the *wild-*type genotypes.

Our study has its strengths and limitations. A main strength of this study is adjusting the measure of effect to factors that were known to increase the risk of gastrointestinal bleeding such as comedication with non-steroidal anti-inflammatory drugs, proton-pump inhibitors, anticoagulants, and previous history of bleeding or peptic ulcer. Memory bias was reduced by demonstrating prompt cards of the most frequent aspirin commercial boxes to the participants. The exclusion of non-white patients also allowed to prevent bias due to racial differences between populations. Moreover, conducting the study in biologically unrelated patients, exclusively, avoided ascertainment bias due to over-representation of SNPs within families (Malomane et al., 2018). Nonetheless, due to the genetic differences between races, our results cannot be generalized to populations that are not Caucasian. Another major limitation of our study is the insufficient sample size. This caused the 95% confidence intervals of RERI and S to be very wide, and consequently the null hypothesis could not be rejected. This occurred despite the fact that we present the largest case-control on this topic. Indeed, low statistical power is a common limitation across candidate gene studies (Salanti et al., 2005; Dumas-Mallet et al., 2017). We therefore consider that our findings should be treated as preliminary ones and they should be further confirmed by (1) other studies with larger sample size before their application in clinical settings; and (2) meta-analysis of other studies about modification effect from aspirin-SNPs interaction on UGIH. Due to the limited sample size, we were not able to determine the dose-response relationship as well as the effect of some SNPs. In addition, we could not analyze the effect of carrying more than one SNP on aspirin-related UGIH due to the limited number of cases and/or controls in some combinations of aspirin exposure and genetic profile (data not shown). In spite of these limitations this study can help designing future pharmacogenomic ones in order to better understand the interaction effect between genetics and aspirin use on UGIH.

In conclusion, our preliminary findings suggest that certain genetic variations might modify the risk magnitude of aspirin-related UGIH. Future studies with larger sample size and additional gene expression analyses are needed to confirm our results and explain the biological effect of these SNPs on aspirin-related UGIH. If confirmed, these findings would have high impact at the clinical and public health levels, because they will permit personalized aspirin prescriptions to prevent diseases with high incidence and mortality rates.

## Data Availability Statement

The datasets presented in this study can be found in online repositories. The names of the repository/repositories and accession number(s) can be found below: https://doi.org/10.6084/m9.figshare.12030606.v1.

## Ethics Statement

The studies involving human participants were reviewed and approved by Comité Ético de Investigación Clínica, Área De Salud Valladolid - ESTE (CEIC-VA-ESTE-HCUV; protocol number: PI-14-142) Comité Ético de Investigación Clínica De Galicia (CEIC-G; protocol number: 2013/263), Comité Ético de Investigación Clínica De Euskadi (CEIC-E; protocol number: PI2013101), and Comité Ético de Investigación Clínica De Barcelona (CEIC; protocol number: Es38121226Z). The patients/participants provided their written informed consent to participate in this study.

## Author Contributions

CA, LI, XV, and AF conceived the research idea, designed the study and supervised and administered the project. NM did the literature review and conceptualized and wrote the manuscript. NM and MP-L analyzed the data. NM interpreted the data and participated in the genetic laboratory testing. AF supervised the data analysis and interpretation. MZ-C, EI-G, IP–Z, FM-G, JD-M, LV, LM-A, MS-G, and VV-G were involved in patients’ recruitment and data registration. All authors contributed to the article and approved the submitted version.

## Funding

This work was supported by a grant from Instituto de Salud Carlos III (PI12/02414)/Plan Estatal de I+D+I 2012-2016; Fondo Europeo de Desarrollo Regional (FEDER); the Novartis, Pfizer and Dr Esteve pharmaceutical companies; the Health Research Fund/Fondo de Investigación Sanitaria (PI021512, PI021364, PI020661, and PI021572); Ministry of Health & Consumer Affairs, Spain (SAF2002-04057); Galician Regional Authority, Spain (PGIDIT03PXIC20806PN); Department of Health of the Basque Country (03/11092 and 11/111103); Fundacion Vasca de innovacion e investigacion sanitarias (OSIBG19/002 and OSIBG18/105). The genotyping service was carried out at CEGEN-PRB3-ISCIII; Instituto de Salud Carlos III and ERDF (PT17/0019, of the PE I+D+i 2013-2016).

## Conflict of Interest

The authors declare that the research was conducted in the absence of any commercial or financial relationships that could be construed as a potential conflict of interest.
