# Corrigendum: Influence of Polymorphisms Involved in Platelet Activation and Inflammatory Response on Aspirin-Related Upper Gastrointestinal Bleeding: A Case-Control Study

**DOI:** 10.3389/fphar.2020.01072

**Published:** 2020-07-17

**Authors:** Narmeen Mallah, Maruxa Zapata-Cachafeiro, Carmelo Aguirre, Eguzkiñe Ibarra-García, Itziar Palacios-Zabalza, Fernando Macías-García, J. Enrique Domínguez-Muñoz, María Piñeiro-Lamas, Luisa Ibáñez, Xavier Vidal, Lourdes Vendrell, Luis Martin-Arias, María Sáinz-Gil, Verónica Velasco-González, Adolfo Figueiras

**Affiliations:** ^1^ Department of Preventive Medicine, University of Santiago de Compostela, Santiago de Compostela, Spain; ^2^ Consortium for Biomedical Research in Epidemiology and Public Health (CIBER en Epidemiología y Salud Pública - CIBERESP), Carlos III Health Institute, Madrid, Spain; ^3^ Biocruces Bizkaia Health Research Institute, Pharmacotherapy Group, Bizkaia, Spain; ^4^ University Hospital of Galdakao-Usansolo. Basque Country Pharmacovigilance Unit, Osakidetza, Spain; ^5^ Pharmacology Department, Medicine and Nursing Faculty, University of the Basque Country, Barakaldo, Bizkaia, Spain; ^6^ Osakidetza Basque Health Service, Urduliz Hospital, Pharmacy Department, Urduliz, Spain; ^7^ Department of Gastroenterology and Hepatology, University Hospital of Santiago de Compostela, Santiago de Compostela, Spain; ^8^ Health Research Institute of Santiago de Compostela (IDIS), Santiago de Compostela, Spain; ^9^ Department of Pharmacology, Therapeutics and Toxicology, Catalonian Institute of Pharmacology, Clinical Pharmacology Service, Vall d’Hebron University Teaching Hospital, Autonomous University, Barcelona, Spain; ^10^ Centre for Research on Drug Safety (CESME), Valladolid University, Valladolid, Spain

**Keywords:** aspirin, upper gastrointestinal haemorrhage, genetic polymorphism, pharmacogenomics, platelet, interaction

In the original article, there was an error. The title lacks the first two words “Influence of.” A correction has been made to the title: *Influence of Polymorphisms Involved in Platelet Activation and Inflammatory Response on Aspirin-Related Upper Gastrointestinal Bleeding: A Case-Control Study*.

Furthermore, the name of one of the authors was incorrectly spelled in the **References** section as “Throat and Cuzick, 2015.” It should be “Thorat and Cuzick, 2015.”

Finally, in the original article, there was a typing error. A correction has been made to the section **Results**, subsection **Study Population**, Paragraph 1:

“Of 5,896 interviewed subjects, 326 cases and 748 controls were eligible to be included in the final analysis. The most common exclusion criteria were ineligible endoscopic diagnosis (1,590 cases) and unavailability of biological material (328 cases and 749 controls). The flow diagram of cases and controls enrollment in the study and the reasons of exclusion are presented in supplementary data online (see **Figure S1** and **Table S1** of the supplementary materials attached to this article). **Table 1** summarizes the demographic and clinical characteristics of the study population.”

The authors apologize for these errors and state that they do not change the scientific conclusions of the article in any way. The original article has been updated.

