# Supplementary material for: Influence of Polymorphisms Involved in Platelet Activation and Inflammatory Response on Aspirin-Related Upper Gastrointestinal Bleeding: A Case-Control Study
Source: Front Pharmacol. 2020 Jun 9;11:860. doi: 10.3389/fphar.2020.00860 (PMC7325915; doi:10.3389/fphar.2020.00860)
Supplement: Supplementary file 1 [file DataSheet_1.pdf]

Supplementary Material

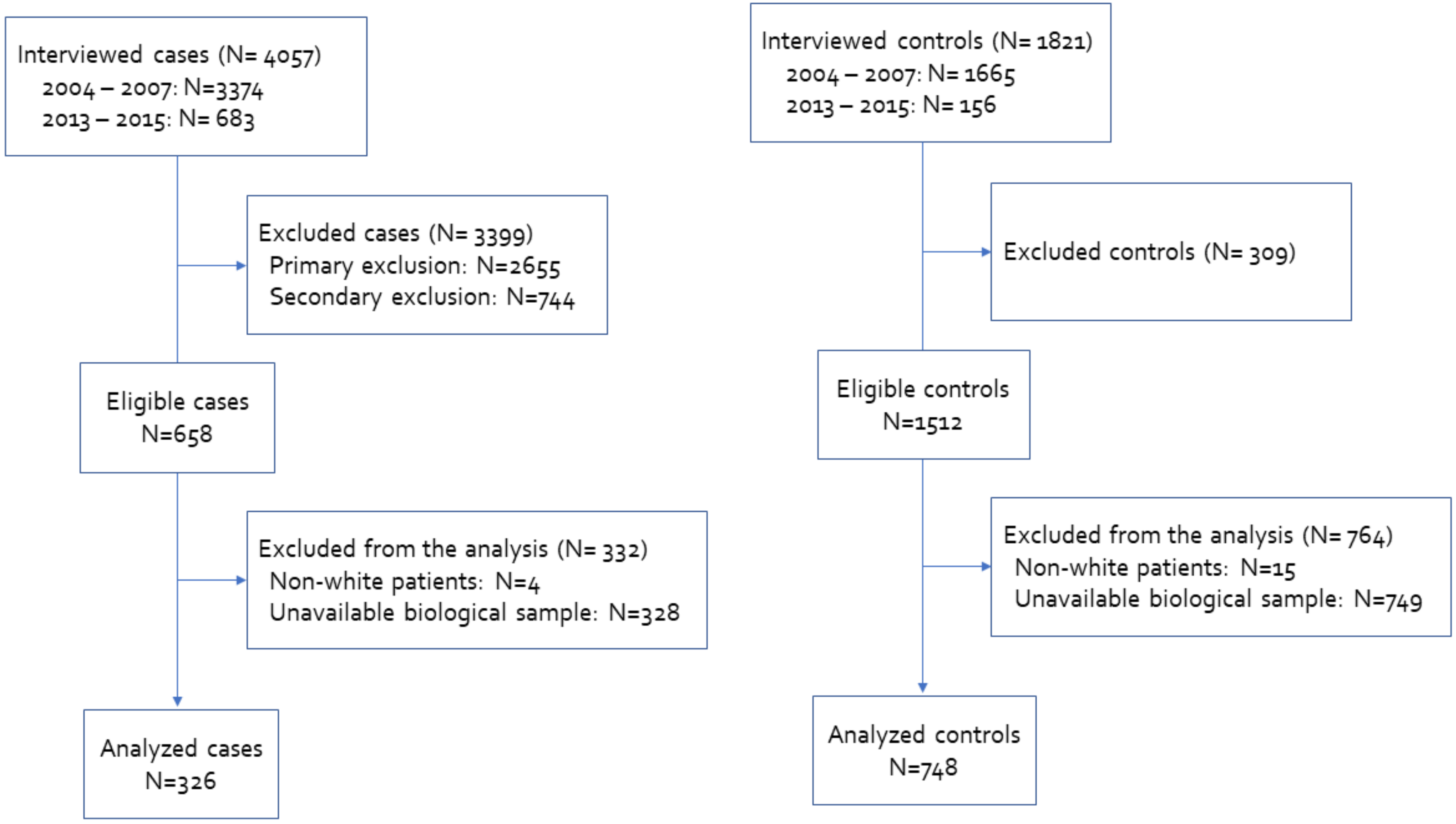

Supplementary Figure 1. Flow of the cases and the controls throughout the two stages of the project

Supplementary Table 1. Motives of exclusion of the cases and controls

Reasons of Exclusion*	EMPHOGEN I (2004-2007)	EMPHOGEN II (2013-2015)
**CASES (N = 3731)**	**3120**	**611**
**Primary Exclusions (N = 2655)**	**2147**	**508**
Age < 18	31	2
Excludable endoscopic diagnosis	1213	377
History of Upper Gastrointestinal Hemorrhage (UGIH)	121	18
Intrahospital UGIH	89	5
UGIH without endoscopic or surgical diagnosis from admission to discharge	121	3
Nasogastric or percutaneous tube carrier	75	2
Less than 3 months residence in study area or Do not belong to the study area	42	7
Admission time < 24 h	208	8
Admission not due to UGIH	154	80
Death	0	2
Other	93	4
**Secondary Exclusions (N = 744)**	**646**	**98**
Refusal to sign informed consent form	21	0
Occurred at weekend or vacations period	57	21
Death	11	2
Endoscopy performed more than 48 h after admission	83	39
Discharge from hospital or visit to healthcare facility in the 15 days prior to admission	54	20
Severe condition	7	1
Psychological disorders	12	4
Illiterate	2	0
Deaf or blind	1	0
Lives in a residence or closed institution and does not know the drugs taken	7	1
Refusal to answer or failure to complete the interview	12	5
Impossible to conduct interview within the 15-day period preceding admission	6	4
Admission time < 24 h	0	1
Other	373	0
**Excluded from analysis (N = 332)**	**327**	**5**
Non-white patients	4	0
Unavailable biological material	323	5
**CONTROLS (N=1073)**	**1071**	**2**
Refused to sign informed consent form	45	0
Age < 18	1	0
History of UGIH	11	1
Intrahospital UGIH	89	0
Nasogastric or percutaneous tube carrier	2	0
Less than 3 months residence in study area	1	0
Severe condition	1	0
Psychological disorders	1	0
Deaf or blind	3	0
Refusal to answer or failure to complete the interview	80	0
Impossible to conduct interview within the 15-day period preceding admission	60	0
Date of last admission	0	1
Other	13	0
Non-white patients	15	0
Unavailable biological material	749	0

(*): Cases and controls were excluded upon presenting one or more exclusion criteria

Supplementary Table 2. Hardy-Weinberg Equilibrium (HWE) test of the 27 studies SNPs

Gene	Single Nucleotide Polymorphism Reference Number	Genotypes	Cases (N)	Controls	HWE P-value
GP1BA, glycoprotein Ib platelet subunit alpha	rs2243086	GG	223	473	0.821
		GT	91	245	
		TT	11	29	
TBXA2R, thromboxane A2 receptor	rs1131882	AA	11	14	0.067
		AG	87	225	
		GG	228	509	
ADRA2A, Alpha-2A-adrenergic receptor	rs4311994	CC	238	528	0.892
		CT	84	202	
		TT	4	18	
CDKN2B-AS1, CDKN2B antisense RNA 1	rs10120688	AA	92	235	0.140
		AG	168	350	
		GG	66	163	
IL1RN, interleukin 1 receptor antagonist	rs4251961	CC	44	108	0.70
		CT	145	360	
		TT	137	280	
F13A1, coagulation factor XIII A chain	rs3778355	CC	96	229	0.824
		CG	164	373	
		GG	66	146	
PTGS1, prostaglandin-endoperoxide synthase 1	rs1330344	CC	10	25	0.147
		CT	125	259	
		TT	191	464	
PTGS2, prostaglandin-endoperoxide synthase 2	rs5275	AA	148	331	0.561
		AG	139	334	
		GG	22	76	
DPP6, dipeptidyl peptidase like 6	rs1387180	AA	175	416	0.561
		AG	135	288	
		GG	16	44	
TBXA2R, thromboxane A2 receptor	rs2238631	CC	229	521	0.694
		CT	89	205	
		TT	9	22	
TNF, tumor necrosis factor	rs1799964	CC	16	37	0.363
		CT	127	280	
		TT	183	431	
F13B, coagulation factor XIII B chain	rs2990510	GG	31	71	0.450
		GT	128	334	
		TT	167	343	
AGT, angiotensinogen	rs5050	GG	12	34	0.427
		GT	109	234	
		TT	205	480	
PTGS2, prostaglandin-endoperoxide synthase 2<br>PACERR, PTGS2 antisense NFKB1 complex-mediated expression regulator RNA	rs689466	CC	16	41	0.195
		CT	103	241	
		TT	207	465	
NOS3, nitric oxide synthase 3	rs1799983	GG	117	289	0.816
		GT	159	348	
		TT	50	109	
PLA2G7, phospholipase A2 group VII	rs7756935	AA	188	417	0.216
		AC	116	274	
		CC	22	57	
LOC101928516 : Intron Variant<br>LOC105377858 : Intron Variant	rs2502488	AA	12	23	
		AG	84	205	0.601
		GG	230	520	

Supplementary Table 2. Hard-Weinberg Equilibrium (HWE) test of the 27 studies SNPs (continued)

Gene	Single Nucleotide Polymorphism Reference Number	Genotypes	Cases (N)	Controls	HWE P-value
TNF, tumor necrosis factor	rs1800629	AA<br>AG<br>GG	1<br>74<br>251	22<br>172<br>554	0.074
TNF, tumor necrosis factor	rs361525	AA<br>AG<br>GG	3<br>36<br>287	2<br>81<br>665	1.00
IL1B, interleukin 1 beta	rs1143627	AA<br>AG<br>GG	145<br>145<br>36	327<br>326<br>95	0.332
IL1B, interleukin 1 beta	rs16944	AA<br>AG<br>GG	36<br>144<br>146	94<br>327<br>327	0.374
PTGS1, prostaglandin-endoperoxide synthase 1	rs3842787	CC<br>CT<br>TT	292<br>34<br>0	672<br>74<br>2	1.00
PTGS1, prostaglandin-endoperoxide synthase 1	rs3842788	AA<br>AG<br>GG	0<br>21<br>305	2<br>55<br>691	0.319
GSR, glutathione-disulfide reductase	rs3779647	CC<br>CT<br>TT	73<br>163<br>90	145<br>391<br>212	0.16
PTGS1, prostaglandin-endoperoxide synthase 1	rs5788	AA<br>AC<br>CC	5<br>80<br>241	14<br>186<br>548	0.881

Supplementary Table 3. Odds ratios (OR) for upper gastrointestinal bleeding associated with aspirin intake and genetic variations

Single Nucleotide Polymorphism (Reference number)	Wildtype genotype		Genetic Variation		OR (95% CI) for any genetic variation within strata of aspirin intake; p-value	RERI (95% CI)	S (95% CI)
	N cases/controls	OR (95% CI); p-value	N cases/controls	OR (95% CI); p-value			
**rs2990510 T>G**							
Aspirin intake (No)	138/295	1.00	124/341	0.77 (0.55 – 1.07) P=0.1230	0.77 (0.55 – 1.08) P=0.1253	0.51 (-5.58 – 6.61)	1.19 (0.15 – 9.42)
Aspirin intake (Yes)	11/6	3.98 (1.24 – 12.76) P=0.0200	13/11	4.26 (1.58 – 11.47) P=0.0041	1.19 (0.14 – 10.46) P=0.8762		
OR (95% CI) for Aspirin intake within strata of genotype; p-value		3.81 (1.14 – 12.77) P=0.0302		5.57 (2.07 – 15.00) P=0.0007			
**rs2502488 G>A**							
Aspirin intake (No)	188/439	1.00	74/197	0.87 (0.60 – 1.26) P=0.4719	0.87 (0.60 – 1.26) P=0.4751	17.46 (-29.80 – 64.73)	8.36 (0.59 – 118.19)
Aspirin intake (Yes)	17/16	3.50 (1.53 – 8.02) P=0.0031	7/1	20.84 (2.16 – 201.49) P=0.0087	5.66 (0.38 – 84.90) P=0.2093		
OR (95% CI) for Aspirin intake within strata of genotype; p-value		3.51 (1.53 – 8.04) P=0.003		26.45 (2.54 – 275.82) P=0.0062			
**rs1800629 G>A**							
Aspirin intake (No)	197/469	1.00	65/167	0.93 (0.63 – 1.37) P=0.7161	0.93 (0.63 – 1.37) P=0.7045	1.44 (-8.15 – 11.02)	1.44 (0.16 – 12.61)
Aspirin intake (Yes)	20/13	4.37 (1.86 – 10.24) P=0.0007	4/4	5.73 (1.19 – 25.75) P=0.0293	1.38 (0.19 – 9.97) P=0.7471		
OR (95% CI) for Aspirin intake within strata of genotype; p-value		4.33 (1.84 – 10.23) P=0.0008		6.75 (1.30 – 34.96) P=0.0229			

Single Nucleotide Polymorphism (Reference number)	Wildtype genotype		Genetic Variation		OR (95% CI) for any genetic variation within strata of aspirin intake; p-value	RERI (95% CI)	S (95% CI)
	N cases/controls	OR (95% CI); p-value	N cases/controls	OR (95% CI); p-value			
**rs361525 G>A**							
Aspirin intake (No)	28/66	1.00	234/570	0.90 (0.53 – 1.55) P=0.7113	0.89 (0.52 – 1.52) P=0.6651	4.52 (-0.74, 9.78)	-9792.91 (NA)
Aspirin intake (Yes)	2/4	1.10 (0.15 – 8.06) P=0.9278	22/13	5.52 (2.12 – 14.37) P=0.0005	52.70 (1.27 – 2182.58) P= 0.0369		
OR (95% CI) for Aspirin intake within strata of genotype; p-value		0.55 (0.05 – 6.44) P=0.6324		6.08 (2.64 – 14.00) P<0.0001			
**rs1143627 G>A**							
Aspirin intake (No)	122/270	1.00	140/366	0.81 (0.58 – 1.13) P=0.2116	0.81 (0.58 – 1.13) P=0.2089	13.56 (-5.78 – 32.89)	32.63 (0.46 – 2313.17)
Aspirin intake (Yes)	8/13	1.62 (0.57 – 4.64) P=0.3676	16/4	14.99 (4.11 – 54.62) P<0.0001	120.26 (2.08 – 6942.25) 0.0206		
OR (95% CI) for Aspirin intake within strata of genotype; p-value		1.76 (0.59 – 5.24) P=0.307		16.95 (4.57 – 62.79) P<0.0001			
**rs16944 A>G**							
Aspirin intake (No)	123/270	1.00	139/366	0.80 (0.57 – 1.12) P=0.1981	0.80 (0.57 – 1.12) P=0.1948	13.53 (-5.74 – 32.80)	33.29 (0.43 – 2550.99)
Aspirin intake (Yes)	8/13	1.62 (0.57 – 4.63) P=0.3703	16/4	14.95 (4.10 – 54.46) P<0.0001	120.26 (2.08 – 6942.25) P=0.0206		
OR (95% CI) for Aspirin intake within strata of genotype; p-value		1.75 (0.59 – 5.20) P=0.3149		16.99 (4.59 – 62.83) P<0.0001			

Single Nucleotide Polymorphism (Reference number)	Wildtype genotype		Genetic Variation		OR (95% CI) for any genetic variation within strata of aspirin intake; p-value	RERI (95% CI)	S (95% CI)
	N cases/controls	OR (95% CI); p-value	N cases/controls	OR (95% CI); p-value			
**rs3842787 C>T**							
Aspirin intake (No)	23/65	1.00	239/571	1.47 (0.82 – 2.62) P=0.1966	1.46 (0.81 – 2.61) P=0.2049	4.13 (-4.42 – 12.70)	2.77 (0.18 – 42.84)
Aspirin intake (Yes)	3/2	2.87 (0.33 – 24.78) P=0.3382	21/15	7.47 (2.83 – 19.71) P<0.0001	2.98 (0.15 – 57.41) P=0.4703		
OR (95% CI) for Aspirin intake within strata of genotype; p-value		6.32 (0.30 – 134.09) P=0.2368		5.08 (2.26 – 11.41) P=0.0001			
**rs3842788 G>A**							
Aspirin intake (No)	246/591	1.00	16/45	0.78 (0.40 – 1.52) P=0.4724	0.79 (0.40 – 1.53) P=0.4777	2.96 (-12.96 – 18.01)	1.94 (0.14 – 27.12)
Aspirin intake (Yes)	21/15	4.35 (1.94 – 9.75) P=0.0004	3/2	7.10 (0.89 – 56.61) P=0.0643	1.23 (0.12 – 12.64) P=0.8595		
OR (95% CI) for Aspirin intake within strata of genotype; p-value		4.53 (2.02 – 10.19) P=0.0003		9.78 (0.88 – 108.56) P=0.0633			
**rs5788 C>A**							
Aspirin intake (No)	197/464	1.00	65/172	0.93 (0.63 – 1.37) P=0.7133	0.93 (0.63 – 1.37) P=0.7163	3.55 (-8.52 – 15.62)	2.21 (0.26 – 18.46)
Aspirin intake (Yes)	17/14	4.00 (1.68 – 9.51) P=0.0017	7/3	7.48 (1.56 – 35.86) P=0.0119	2.55 (0.29 – 22.05) P=0.3958		
OR (95% CI) for Aspirin intake within strata of genotype; p-value		4.52 (1.82 – 11.21) P=0.0011		7.55 (1.60 – 35.61) P=0.0106			

(*): Odds Ratio adjusted for: period of patients´ recruitment, previous history of arthrosis, infection with *Helicobacter pylori*, gastrointestinal disorders (ulcer and bleeding), exposure to non-steroidal anti-inflammatory drugs except aspirin, exposure to inhibitors of the proton pump, exposure to antiaggregant, exposure to anticoagulants, and the interview variables (the number and the reliability of the interview).
